# Tumor Gene Expression Profiling in Women with Breast Cancer: Test Category: Prognostic

**DOI:** 10.1371/currents.RRN1178

**Published:** 2010-09-02

**Authors:** Cecelia Bellcross, W. David Dotson

**Affiliations:** ^*^National Office of Public Health Genomics, CDC and ^†^Office of Public Health Genomics, CDC

## Abstract

Differences in the expression of specific genes within breast tumors have been associated with risk of recurrence after treatment. Most women with Stage I or II node-negative breast cancer (especially when estrogen-receptor positive and treated with tamoxifen) remain disease-free at 10 years. Information on risk of recurrence could help identify women most likely to benefit from chemotherapy. Several clinically available gene expression profiles (GEP) provide “recurrence risk scores” that are intended to supplement information used by clinicians and patients in treatment decision-making.

## **Clinical Scenario**

Tumor gene expression profiling in women with Stage I or II node-negative breast cancer to predict recurrence risk and guide decisions about chemotherapy.

## **Test Description**

Reverse transcription PCR is used by Oncotype DX and the H:I Ratio Test (Breast cancer Gene Expression Ratio Assay) for the detection and quantification of mRNA in formalin fixed, paraffin-embedded breast cancer tissue. Oncotype DX analyzes the expression of 21 genes (16 cancer related and 5 normative). The H:I Ratio Test measures the ratio of the expression of the homeobox gene-B13 (HOXB13) and the interleukin-17B receptor gene (IL17BR). MammaPrint uses micro-array technology to test for 70 cancer related and about 1800 normative genes in unfixed (fresh or frozen) breast cancer tissue. [1] [2]

## **Public Health Importance**

Breast cancer is the most commonly diagnosed cancer in U.S. women with an estimated 207,090 new cases in 2010. Among U.S. women, it is the second leading cause of cancer-related deaths (estimated 39,840 deaths in 2010).[3] Stage I/II accounts for over 50% of all diagnoses, and is associated with a 5 year survival rate of 98%.[4] Among women with early stage node-negative disease, the majority elect to receive chemotherapy on the basis of standard recurrence risk classification using tumor characteristics.[5] [6] Approximately 60%, 5%, and 0.5% of women respectively will experience minor, major or fatal toxicity from chemotherapy. [7] [8] Studies of use of breast cancer GEP suggest changes in treatment decisions in approximately 25-30% of cases, most commonly selection of endocrine therapy alone due to reclassification of women from high to low-risk of recurrence .[9] [10] If tumor gene expression profiles are conclusively shown to result in more accurate classification of women into low and high recurrence risk categories, theoretical benefits would include avoidance of unnecessary chemotherapy and reduced disease recurrence rates.

## **Published Reviews, Recommendations and Guidelines**

**Systematic evidence reviews**

Agency for Healthcare Research and Quality, Evidence Report/Technology Assessment [2]Blue Cross and Blue Shield Association, Technology Assessment [11] 

Recommendations by independent group*

Available data have been systematically reviewed and evaluated, providing the basis for a recommendation statement: “The Evaluation of Genomics in Practice and Prevention (EGAPP) Working Group found insufficient evidence to make a recommendation for or against the use of tumor gene expression profiles to improve outcomes in defined populations of women with breast cancer.”[1] 

**Guidelines by professional groups**

- National Comprehensive Cancer Network: Clinical Practice Guidelines in Oncology - Breast Cancer [12]
- American Society of Clinical Oncology: 2007 Update of Recommendations for the use of tumor markers in breast cancer [13]
- ECRI Institute: Policy Statement [14]

* independent groups include the US Preventive Services Task Force (USPSTF) and Evaluation of Genomic Applications in Practice and Prevention (EGAPP) Working Group.

## **Evidence Overview**

***Analytic Validity*:**

Test accuracy and reliability in measuring differences in expression of relevant genes (analytic sensitivity and specificity).

- A 2009 EGAPP Working Group recommendation statement [1] suggested that:

- limited published data are available regarding assay performance of clinically available GEP,
- analytic sensitivity and specificity cannot be estimated because no reference technology (“gold standard”) is available for comparison,
- initial test failure rates ranged from 12-19%, partly because of problems with tissue sampling and processing.

- One published reference on Oncotype DX analytical validity sponsored by the test manufacturer is available. [15]

- The FDA summary of MammaPrint provided information on result and classification accuracy [16]

***Clinical Validity*:**Test accuracy and reliability in predicting breast cancer recurrence and benefit from chemotherapy (predictive value).

- Several studies have reported on the clinical validity for OncotypeDX.[17] [18] [19] [20]
- A recent study reported an association between the Oncotype DX recurrence score and risk of locoregional recurrence using data from the NSABP B-14 and B-20 trials.[21]
- Published studies regarding MammaPrint have suggested a significant association between the MammaPrint Index (high vs. low risk) and 5 and 10 year distant recurrence rates.[22] [23] [24] However:

- efficacy in ER positive vs. negative cases is unclear,[1]
- added value beyond standard risk stratification is unclear.[1]

- A parallel, prospective evaluation was recently published comparing three gene expression profile tests analogous to Oncotype DX, Mammaprint and the H:I Ratio Test.

- All were significantly associated with time to progression; however, concordance of results among the three classifiers was relatively low, classifying only 45-61% of patients in the same category.[25]

***Clinical Utility*: **Net benefit of test in improving health outcomes.

- Retrospective analysis of one arm of a single prospective trial (NSABP B-20) showed that chemotherapy (in addition to tamoxifen) was most beneficial in women in the Onco*type *DX high risk category.[20]
- A prospective multicenter study published in 2010 reported on the impact of Oncotype DX on patient and physician adjuvant chemotherapy decisions.[9]
- Analyses of economic implications of Oncotype DX have been published. [5] [26]
- A 2010 publication explored women’s experiences with genomic testing for breast cancer recurrence risk in terms of how scores were received and understood.[27]
- A 2009 EGAPP recommendation reported that no direct evidence linked the use of MammaPrint or the H:I test to clinical outcomes.[1]
- Ongoing trials include:
  - TAILORx trial (Oncotype DX)[28]
  - MINDACT trial (MammaPrint)[29]

## **Links**

For recent additions to the literature, see PubMed special query (2007-present).

U.S. Food and Drug Administration: Search FDA 510(k) database

Last updated: June 8, 2010
